# Design of a Compact Multicyclic High-Performance Atmospheric Water Harvester for Arid Environments

**DOI:** 10.1021/acsenergylett.4c01061

**Published:** 2024-06-26

**Authors:** Xiangyu Li, Bachir El Fil, Buxuan Li, Gustav Graeber, Adela C. Li, Yang Zhong, Mohammed Alshrah, Chad T. Wilson, Emily Lin

**Affiliations:** † Department of Mechanical Engineering, Massachusetts Institute of Technology, Cambridge, Massachusetts 02139, United States; ‡ Department of Mechanical Aerospace and Biomedical Engineering, University of Tennessee, Knoxville, Tennessee 37996, United States; § Department of Chemistry, Humboldt-Universität zu Berlin, 12489 Berlin, Germany

## Abstract

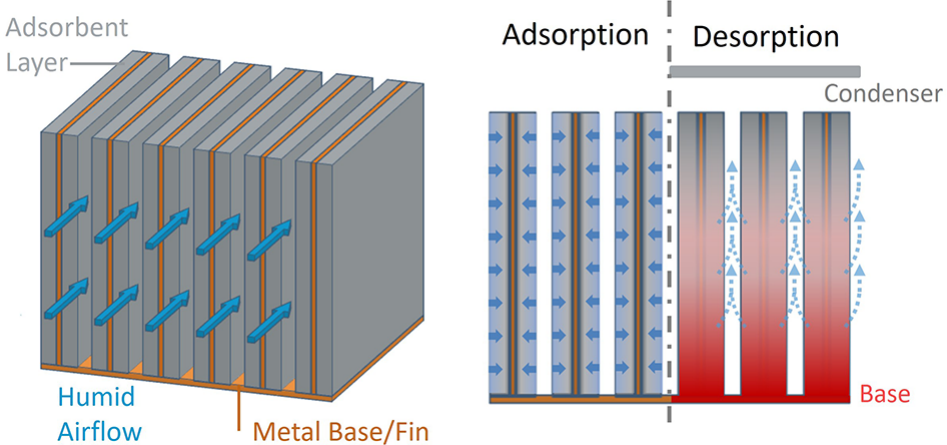

Water scarcity remains a grand challenge across the globe. Sorption-based atmospheric water harvesting (SAWH) is an emerging and promising solution for water scarcity, especially in arid and noncoastal regions. Traditional approaches to AWH such as fog harvesting and dewing are often not applicable in an arid environment (<30% relative humidity (RH)), whereas SAWH has demonstrated great potential to provide fresh water under a wide range of climate conditions. Despite advances in materials development, most demonstrated SAWH devices still lack sufficient water production. In this work, we focus on the adsorption bed design to achieve high water production, multicyclic operation, and a compact form factor (high material loading per heat source contact area). The modeling efforts and experimental validation illustrate an optimized design space with a fin-array adsorption bed enabled by high-density waste heat, which promises 5.826 L_water_ kg_sorbent_^–1^ day^–1^ at 30% RH within a compact 1 L adsorbent bed and commercial adsorbent materials.

About two-thirds of the global population is suffering from water scarcity, and it is estimated that about 40% of the global annual water demand will not be met by 2030, posing further challenges to public health, agriculture, and industrial applications.^1,2^ While desalination is a maturing technology that shows potential to produce large amounts of fresh water in coastal regions, large-scale desalination based on reverse osmosis demands large capital investment, produces a significant amount of brine waste, and requires sufficient seawater supply, while only being viable in developed coastal regions.^3^ However, there is about 1300 trillion liters of fresh water in the atmosphere that could be readily harvested without relying on the existing liquid water supply.^4^ Sorption-based atmospheric water harvesting (SAWH)^5−13^ promises potable water production in extreme arid environments where conventional atmospheric water harvesting solutions such as fog harvesting and dewing are infeasible. Most of the existing SAWH prototypes in literature are solar-driven water harvesting devices (Figure 1A) incorporating the development of novel adsorbent materials,^7,14−17^ including zeolites,^8^ metal–organic framework materials (MOFs),^5,6^ and hygroscopic composite.^9,10^ Kim et al.^5,6^ demonstrated a solar-driven SAWH with a 1 mm thick MOF-801 coating operated at a relative humidity (RH) of 20%. Further optimization of such devices^11^ and the adoption of dual stages^8^ helped recycle the latent heat of condensation to increase the performance of SAWH devices. However, due to the low solar energy density and inconsistent weather conditions, the amount of water harvested from existing prototypes is still inadequate to meet the daily human needs.^18−24^ Shan et al.^9^ have developed a modularized water harvester with scalable, low-cost, and lightweight LiCl impregnated hygroscopic composite (Li-SHC) sorbents. This allows one to increase the productivity of the system through undergoing desorption of several batches. As an alternative to solar energy as the desorption energy source, electricity, produced by photovoltaic panels, counts as one of the higher energy sources for SAWH devices (Figure 1B). This enables multicyclic operations and maximizes the amount of water collection.^7^ However, this comes at a substantial energy cost with the multicyclic desorption process due to the large adsorption enthalpy, also known as desorption enthalpy. Comparably, existing waste heat from various sources can offer low-cost, high-density energy. The performance of current state-of-the-art solar (passive) and active devices is shown in Table 1. Overall, it is critical to leverage high-density waste heat and develop a compact and cost-effective high-performance SAWH device that meets the growing demand for potable water in extreme arid environments.

**Table 1:** Performance Summary of Solar-Driven and High Energy Density-Driven SAWH^25^

	Conventional Passive SAWH^5,6,8,17^	Active (High Energy Density) SAWH^7,9,10,20,22,26^
Desorption Energy Source	Solar	Combustion, Electricity, Waste Heat
Daily Cycles	Single	Multiple
Desorption Temperature	70–100 °C	>100–150 °C
Water Collection per kg of Sorbent	0.1–0.25 L/day	0.57–2.8 L/day
Daily Water Harvested	0.75–60 mL	20–370 mL
Specific Energy Consumption^a^	4.65–6.88 kWh/L	11.15–22.81 kWh/L

a The specific energy consumption (SEC) is calculated based on kWh_th_ (thermal) per liters of water collected daily.

**Figure 1:**
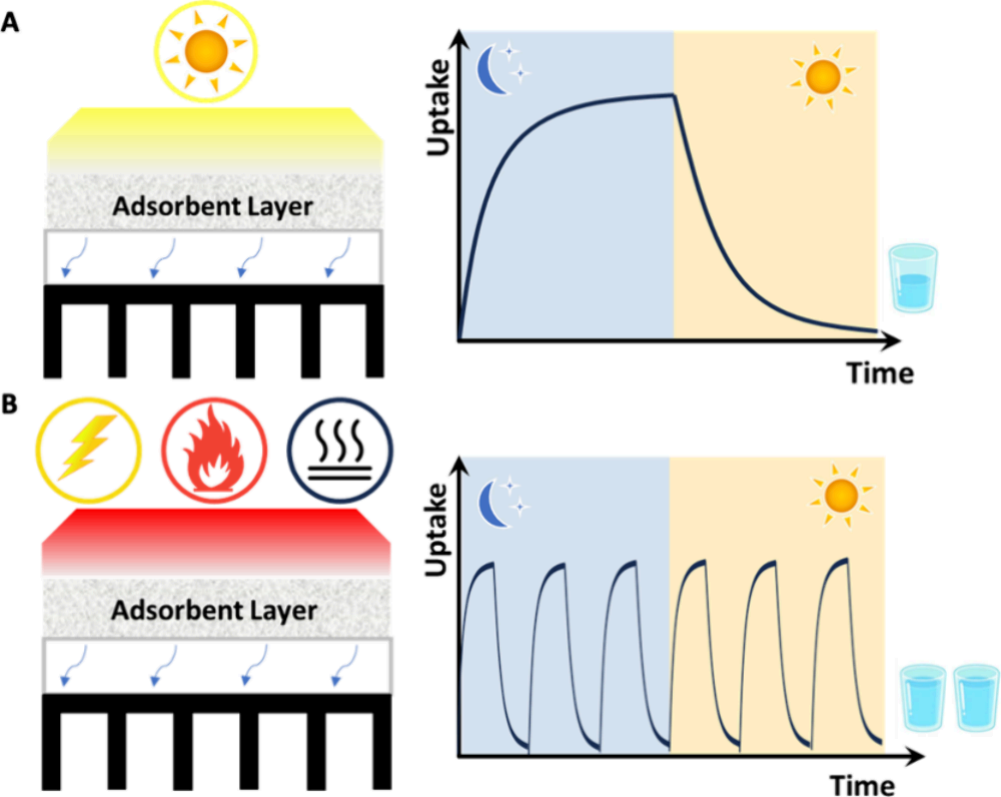
Comparison between solar-driven (passive) and high-energy-driven SAWH devices. (A) SAWH designs driven by solar energy. Limited by solar energy density, the adsorption/desorption cycle often synchronizes with the day–night cycle, resulting in a small amount of daily water production. (B) High-density energy sources to drive SAWH include electricity, biomass combustion, or various sources of waste heat. With high-density energy to drive desorption, multicyclic operation with thinner coatings achieves faster kinetics and higher daily water production.

In this work, we design a compact adsorption bed for an atmospheric water harvesting device driven by high-density waste heat, therefore enabling fast cycling kinetics and maximizing the daily water production. To design a fast-cycling device, it is important to consider both material and device design, which helps in optimizing the multicyclic performance of the device. Material-level water sorption properties, including sorption isotherm, water diffusivity, and adsorption enthalpy *h*_ads_, are the heart of SAWH devices. An ideal sorbent should exhibit a high-water uptake with negligible hysteresis and fast water sorption kinetics and require a minimal amount of energy to regenerate. All of these are essential to design the device where the sorbent will be incorporated. Additionally, material innovations can only be effective when the insights from material science can be organically connected to device realization, although there is still a giant knowledge barrier between them. Device operation is determined by the water transport and energy exchange processes that need to be optimized synergistically by incorporating materials insights. By utilizing an array of thin adsorption fins (∼mm) rather than a single-layer thick coating (∼cm), rapid adsorption cycling is enabled. We identify and optimize several key design parameters associated with air channels between the adsorbent fins to maximize water uptake. Specifically, there is a trade-off between water vapor supply and diffusion resistance for multicycle operation. A thermofluidic numerical model was developed to optimize the novel adsorption bed. With enhanced adsorption kinetics and high-density desorption energy, the proposed design promises high water production within a compact form factor. To validate the numerical model, an experimental prototype of the adsorption bed is fabricated and coated with a commercial adsorbent. We used AQSOA-FAM-Z02 as the adsorbent in all of the experiments due to its low cost and wide availability; however, the work can be readily translated to other adsorbents. AQSOA-FAM-Z02 is based on the (silico)aluminophosphate SAPO-34 zeolite, hereafter referred to as AQSOA-Z02. The prototype (6 cm × 6 cm × 5.5 cm) is used to characterize adsorption and desorption processes, with a volume of 200 mL and total mass of 0.4 kg incorporating 10.85 g of Z02. Both modeling and experimental efforts show that a fin-array adsorbent bed with a compact 1 L form factor promises 1.3 L day^–1^ potable water at 30% RH, while utilizing 230 g AQSOA Z02. Overall, this work provides a design framework for a compact adsorbent bed for an SAWH device driven by a waste heat energy source.

## Adsorption Bed Design and Modeling

**Figure 2:**
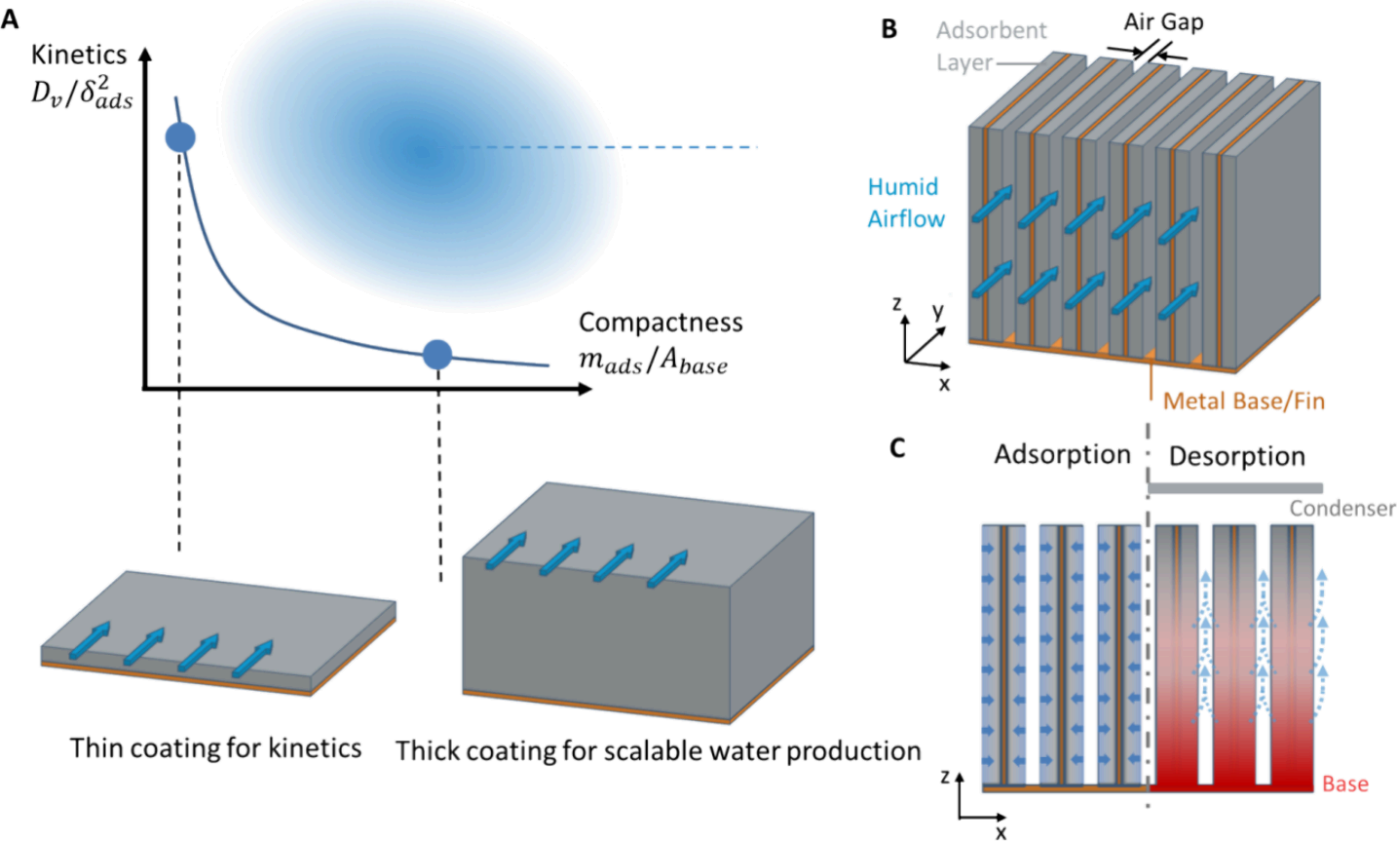
Adsorbent bed design considerations. (A) Optimization of kinetics and compactness for atmospheric water harvesting with single-layer coatings. Humid air flows above the coating surface. Thin coatings enable fast kinetics and multicyclic operation but sacrifice the compactness. (B) Compact adsorbent bed design with an array of adsorbent fins (arranged in the *x*-axis), consisting of metal sheets and adsorbent coatings. Humid air flows through the air gaps (along the *y*-axis) between the adsorbent fins. (C) Front view during the adsorption and desorption processes. During adsorption, humid air enters the air channels and water vapor is adsorbed into the coating, illustrated by blue arrows. During desorption, the sorbent coating is heated by thermal energy input from the base along the *z*-axis. Desorbed water vapor is represented by dashed blue arrows.

State-of-the-art solar-driven SAWH uses a flat adsorbent coating for water adsorption, as depicted in Figure 1A,B. Previous studies often adopted thick adsorption coatings to ensure sufficient and compact sorbent loading while operating a single cycle per day, synchronized with the day–night cycle.^5,8^ The thickness of the coating was determined to optimize the usage of daytime solar energy for desorption processes while harnessing the cooler and higher humidity nighttime conditions for effective adsorption, thus aligning operation with the natural diurnal thermal cycles. This strategy leads to long cycle times and slow kinetics, resulting in a small amount of daily water production, as illustrated in Figure 1A. Recent studies^7,9,27^ explored multicyclic operation (Figure 1B), which utilized thinner coatings for faster kinetics, as well as dual-stage configuration for latent heat recovery to achieve higher energy efficiency.^8,28,29^ Although various device designs have been investigated, overall water production is still significantly limited by passive desorption using solar energy, and the slow kinetics of the adsorbent bed hinders multicyclic operation. With flat single-layer adsorbent coatings fabricated with a dip coating approach, an intrinsic trade-off exists between the cycling kinetics and device compactness, as shown in Figure 2A. The sorbent coating kinetics scale as *D*_v_ /δ_ads_^2^, where *D*_v_ is the effective diffusivity of vapor transport through the coating thickness δ_ads_. On the other hand, compactness is quantified as *m*_ads_/*A*_base_, where *m*_ads_ and *A*_base_ are the adsorbent mass and base projected areas of the adsorbent coating. Alternatively, to maintain both favorable kinetics and compactness (blue region in Figure 2A), we propose a fin-array adsorbent bed (Figure 2B). The fin array (aligned in the *x*-axis) enables faster kinetics due to a thin coating while maintaining a compact form factor. Each adsorbent fin consists of a metal sheet sandwiched between two metal foams. The metal fin enables efficient heat transfer, while the metal foams are coated with sorbent. The fins are aligned in parallel with narrow air gaps in between. Humid air flows along the *y*-axis during the adsorption process. Figure 2C is the front view of the adsorption bed. The blue arrows represent vapor diffusion and adsorption from the humid air flow to the adsorbent coatings, and dashed blue arrows represent desorbed water vapor from the heated adsorbent coating. Due to the thin coatings (∼mm) that are exposed to humid air flow, the enhanced kinetics for adsorption process promise rapid cycling operations. Additionally, by minimizing the air gap thickness between the adsorbent fins, we ensured that a large amount of adsorbent can be packed within a small footprint. Thin air gaps also enhance convective heat transfer, which effectively prevents the overheating of adsorbent coatings. Once the adsorption cycle is completed, the fin base is heated by waste heat for desorption. Thermal energy diffuses from the base along the height of the fins (*z*-axis), leading to a temperature rise and releasing the adsorbed water vapor.

**Figure 3:**
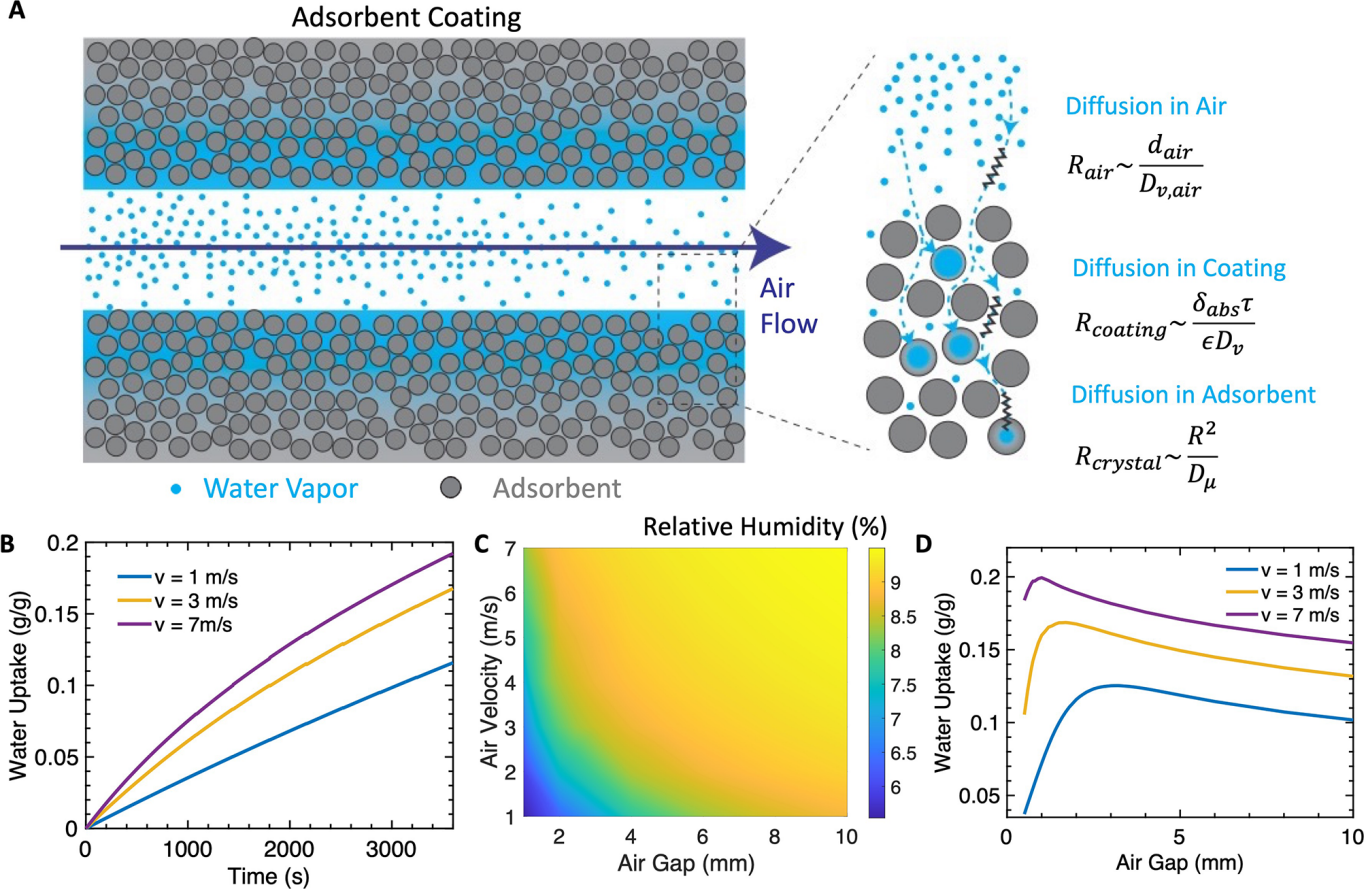
Adsorption simulation results. (A) Water vapor transport path and associated mass transport resistance. (B) Transient water uptake of adsorbent with different air flow velocities. (C) Outlet air flow humidity with different air gap thicknesses and air velocities. (D) Water uptake as a function of different air velocities and air gap thicknesses. Optimized air gaps are identified, as a trade-off between vapor supply and diffusion resistance in the air channels.

During the adsorption process, the mass transport of water vapor from the humid air flows to the adsorbent crystals consisting of three main resistance, *R*_air_, *R*_coating_, and *R*_crystal_, as shown in Figure 3A. The advective transport resistance is neglected due to the high Peclet number with the humid air flow, summarized in Supporting Information Table S1. The vapor transfer from humid air flow to the coating surface is governed by mass diffusion (*R*_air_), determined by the vapor diffusivity and air gap thickness. Within the porous coatings, mass transport is dominated by intercrystalline diffusion (*R*_coating_). Lastly, both particle size and intracrystalline diffusivity affect the transport resistance (*R*_crystal_) into the adsorbent crystals from the adsorbent coating pores. Figure 3B illustrates the effect of air flow velocities on the average water uptake during adsorption. Here, we assumed a simplified Type IV isotherm profile, with a step function at 6% RH. More details on adsorption simulations are included in Note S1 and Figures S1 and S2. Enabled by a higher flow rate, a larger amount of moisture is supplied to the adsorbent, leading to faster overall kinetics, with the trade-off of a higher pressure drop. Figure 3C shows the outlet relative humidity at different air flow velocities and air gaps after a 30 min adsorption cycle. The vapor supply can restrain the adsorption rate, leading to lower outlet humidity at low air flow velocities or small air gaps, as shown in Figure 3D. With an arid ambient condition (RH = 10%), there is a minimal air gap needed to ensure sufficient vapor supply based on air flow velocities. However, as the air gap continues to increase, the adsorption rate drops, resulting in an optimized air gap where a maximized adsorption rate is achieved. An air gap too large will reduce the water vapor diffusion rate to the coating surface since the vapor transport rate perpendicular to the air flow is inversed proportional to *d*_air_^2^. A scaling analysis is provided in Note S2 and Figure S5. Therefore, a larger air gap benefits the vapor supply but has a negative impact on *R*_air_ and the vapor transport rate. This shows that there exists an optimal air gap thickness, which is a critical design parameter to maximize the adsorption potential of the materials. This is contrary to traditional thoughts that larger air gaps are preferred with a greater vapor supply.

**Figure 4:**
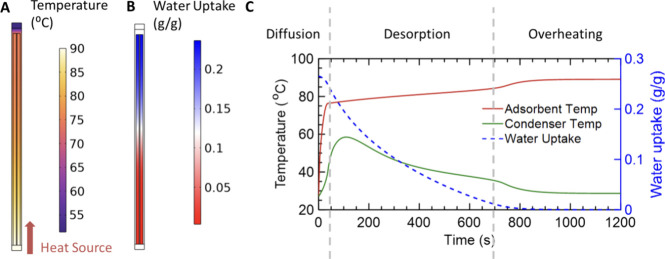
Desorption simulation results. (A) Temperature profile and (B) adsorbent water uptake profile at 300 s after the start of desorption. (C) Temperature and water uptake response during the desorption operation. Red and green curves represent the average temperatures of adsorbent and condenser, and the blue dashed curve represents water uptake in the adsorbent. In the first 50 s, thermal energy diffuses through the fins, before the adsorbent reaches its desorption temperature. As the temperature increases, water vapor is desorbed, increasing the condenser temperature and reducing the water uptake. Due to the constant heat flux, the desorption rate is mostly linear, until the adsorbent is further heated at the end of desorption.

Figure 4 illustrates the desorption simulation with a constant temperature of 90 °C to simulate the high-density waste heat (see Figures S3 and S4 for more information). Panels A and B of Figure 4 show the temperature and water uptake profiles at 300 s. Due to the high adsorption enthalpy (∼3420 kJ kg^–1^ for AQSOA Z02)^30^ and high thermal conductivity of the adsorbent fin, there is minor temperature variation in the adsorbent fin. The desorption initiates at the fin base and continues toward the fin tip, as thermal energy travels along the fin height. The transient temperature and water uptake responses are shown in Figure 4C. Gray dashed lines highlight three main phases. From the start up to 50 s, thermal diffusion dominates before the adsorbent reaches its desorption temperature. From 50 to 700 s, the applied thermal energy desorbs the water vapor out from the adsorbent. Once desorption is close to completion at around 700 s, the thermal energy starts to contribute to the sensible heating, which leads to adsorbent saturation to the base temperature. In this design, desorption occurs within a closed system, i.e., the sorbent bed is isolated from the ambient air, to ensure that the desorbed water vapor is contained in the system to maximize the amount of water condensate. More detailed adsorbent bed optimization is shown in Note S3 and Figures S6–S8.

## Adsorption and Desorption Experimental Testing

**Figure 5:**
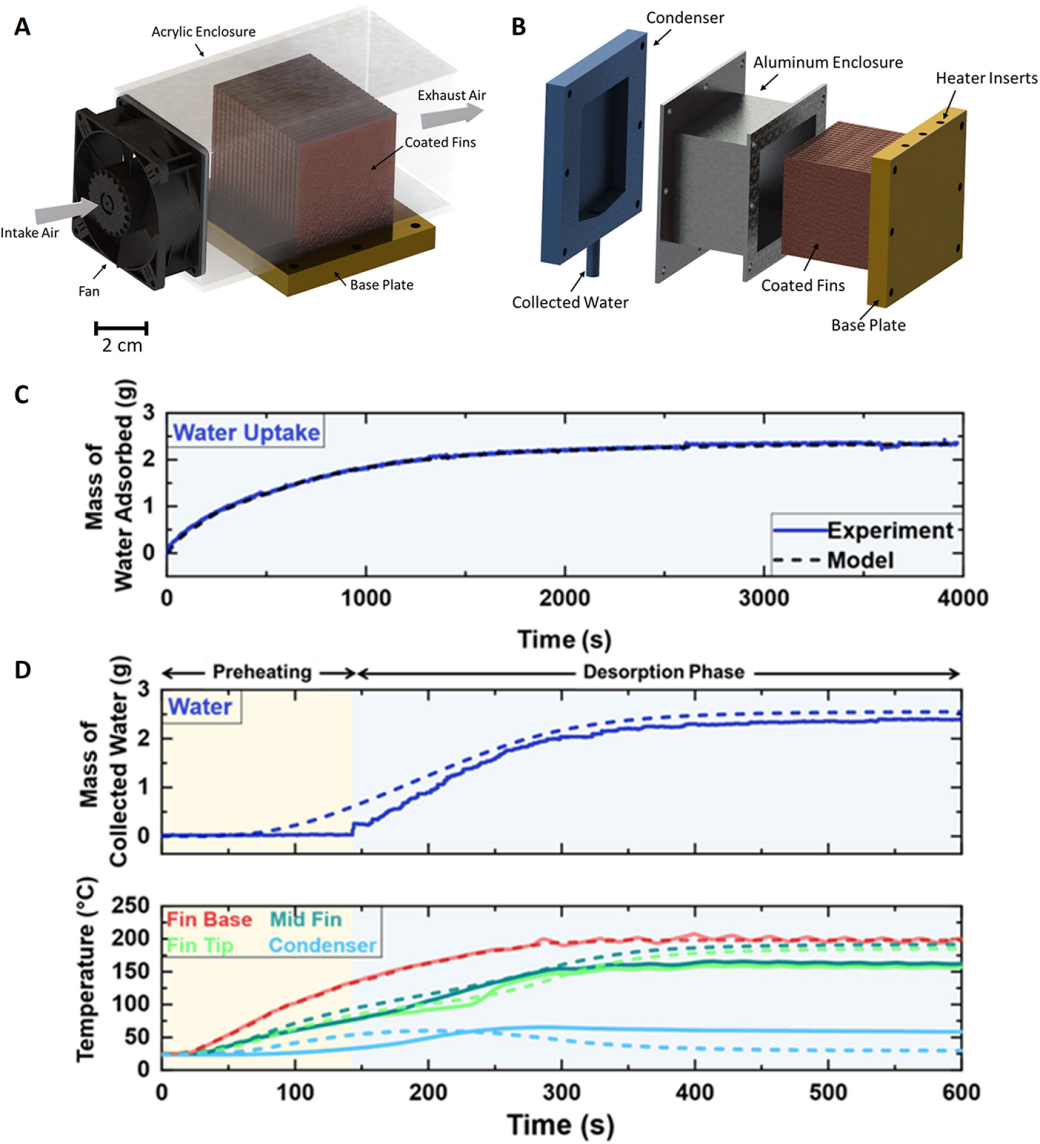
Experimental validation of adsorption and desorption for AQSOA Z02 adsorption bed. (A) Experimental validation setup of adsorption process, where water adsorption is monitored with weight. (B) Experimental validation setup of the desorption process in an enclosed system, where heat is provided with cartridge heaters. Experimental results are compared with numerical models for (C) adsorption and (D) desorption processes. Experimental and modeling adsorption data are represented by blue solid line and black dashed line, respectively.

In this study, we conceived a proof-of-concept experimental setup with the aim of comprehensively characterizing both the adsorption and desorption processes. Specifically, we devised two smaller-scale experimental configurations (Figure 5A,B), to experimentally validate the numerical thermofluidic models developed for the analysis of adsorption and desorption phenomena, respectively. Within each of the experimental setups, we assembled fin structures on a copper base plate. Each fin unit consisted of a copper sheet with a thickness of 0.15 mm, sandwiched between two layers of copper foams (coating thickness, δ_ads_, of 0.6 mm and a porosity, ε ≥ 0.98). The adsorbent fins had a length (*L*) of 35 mm and a width (*W*) of 40 mm, with a 2 mm air gap separating them.

In the context of the adsorption setup, as elucidated in Figure 5A, an acrylic enclosure was used to guide the fan air intake throughout the adsorption bed. The entire assembly was placed on top of a high-precision mass balance, facilitating quantification of the mass of water vapor adsorbed from the surrounding atmosphere as a function of time. On the other hand, the desorption setup, as shown in Figure 5B, has three cartridge heaters, each with a maximum power capacity of 60 W and J-Type thermocouples attached. The temperature of the copper block was controlled through a proportional-integral derivative (PID) temperature controller. During desorption, an aluminum enclosure was used to contain the adsorption bed. To minimize the dissipation of thermal energy, the aluminum enclosure was insulated. Three J-Type thermocouples were positioned at the base, mid-point, and tip of an adsorbent fin, respectively, thereby measuring a comprehensive temperature profile across the entire fin length. To reduce heat transport from the copper base to the condenser and to prevent any vapor leakage from the experimental apparatus, a high-temperature silicon gasket was placed between the aluminum enclosure and the condenser. Furthermore, the condenser temperature was measured throughout the desorption experiment. The resulting condensate was collected within a graduated cylinder and placed on a mass balance. This configuration facilitated continuous quantification of the mass of accrued water over time.

The experimental setups are illustrated in Figure 5A,B, where the temperature, humidity, and mass data were collected. Detailed information on the experimental setup is included in Note S4 and Figures S9–S11. Numerical simulations were also conducted based on the adsorbent isotherm of Z02 and kinetics. Both experimental characterization and numerical simulation agree well to demonstrate the rapid kinetics, which enables saturated adsorption within 30 min, as shown in Figure 5C, contributed by the fin-array adsorbent bed design. A nominalized weight is defined as actual water adsorbed over the saturated water uptake based on material isotherms. Given available waste heat from biomass combustion or transportation vehicles, the desorption process can be significantly reduced to less than 10 min (Figure 5D) due to its high energy density. Solid lines represent experimental temperature characterization and collected water mass, and dashed lines are simulated from numerical models. The deviation of fin temperatures is due to the possible heat loss of the system, with more analysis in Note S4. A delay was observed between the vapor generation from modeling and liquid water collection from experiment due to the time delay of condensed water flowing to the collecting vessel, which also contributed to the slight offset in the condenser temperature. Based on the modeling and experimental results, the proposed adsorption bed design promises more than 24 cycles per day of water adsorption with high-density waste heat. This translates to ∼1.3 L potable water per day with 0.23 kg of zeolite AQSOA Z02 and a 1 L adsorbent bed, or 2–5 times the daily water production compared to that of previous devices.^25,31^

**Figure 6:**
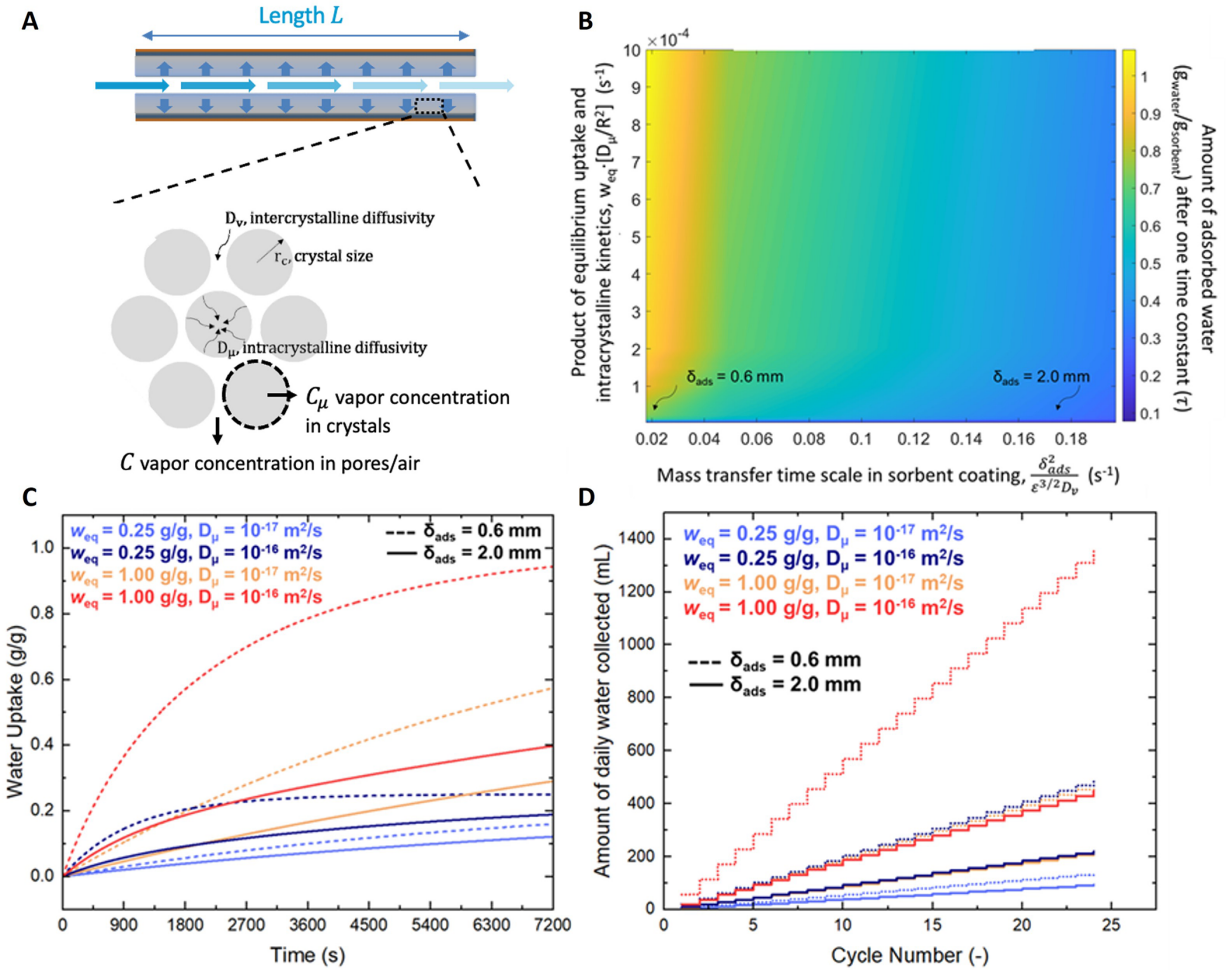
Water sorption and kinetics in the fin-structured adsorbent bed. (A) Schematic showing humid air flowing through the air gap between adsorbent coatings. (B) Design map showing the water uptake as a function of the product of equilibrium water uptake of the adsorbent and its kinetics, *w*_eq_*D*_μ_/*R*^2^, and the time scale of mass transfer in the sorbent coating . (C) Water uptake as a function of time as a function of equilibrium uptake, intracrystalline diffusivity, and coating thickness. (D) Cumulative amount of daily water collection for different sorbent characteristics and thicknesses.

This proposed compact adsorption bed design provides a reliable and effective source of potable water production that can be integrated into various existing applications, such as in buildings, industrial plants, and transportation with suitable high-density waste heat. Further development and implementation of other novel materials can benefit and improve upon the above performance, including metal–organic frameworks and salt-impregnated hybrid materials that achieve higher water uptake and more accessible desorption temperature.^15^ Water vapor transport inside packed sorbent coatings directly influences the kinetics of water capture and release. Generally, the sorption kinetics are governed by multistep water transport processes, including interface resistance (*R*_air_), diffusion resistance in the coating, i.e., intercrystalline resistance through the coating (*R*_coating_), and diffusion within the crystal, i.e., intracrystalline resistance (*R*_crystal_) as depicted in Figure 3A. Typically, significant decline in sorption and desorption kinetics could be a bottleneck for all kinds of large-scale packed sorbents, which mainly tends to increase the diffusion resistances of water vapor transport within the coating, *R*_coating_. To overcome this challenge, we designed a fin-based sorbent bed (Figure 6A) that maintains favorable kinetics while providing a similar water uptake capacity of the sorbent bed. Two fundamental key properties dictate the sorbent’s performance: (1) equilibrium water uptake (*w*_eq_) and (2) kinetics given by the ratio of intracrystalline diffusivity to the square of the particle radius (*D*_μ_/*R*^2^). The ideal sorbent for a multicycle atmospheric water harvesting device would have high uptake and fast kinetics, i.e., maximizing the product of the latter two factors (*w*_eq_·*D*_μ_/*R*^2^). However, as the thickness of the sorbent layer increases, the effect of *R*_coating_ () becomes more pronounced, where δ_ads_ is the thickness of the sorbent layer, ε is the coating packing porosity, τ is tortuosity estimated by random packing porosity ∼ε ^–1/2^, and *D*_v,air_ is the vapor diffusivity in air (∼2.6 × 10^–5^ m^2^ s^–1^).^32^Figure 6B shows the heat map of the instantaneous amount of water uptake after a one-time constant (∼63.2% of the steady state uptake) as a function of intrinsic sorbent properties, i.e., *w*_eq_*D*_μ_/*R*^2^(*y*-axis) and intercrystalline mass transfer time scale  on the *x*-axis. Figure 6B clearly illustrates the importance of the sorbent coating, especially in regions where the product of equilibrium uptake and kinetics is less than 2 × 10^–4^ s^–1^. As the coating thickness increases, instantaneous water uptake into the coating layer decreases. It highlights the significance of the intercrystalline mass transfer resistance and its effect on water capacity. Typically, the ability to tune the sorbent could be difficult and limited; however, significant performance improvement exists just by optimizing the design of the adsorbent bed. As described earlier, enhancing the coating performance could be achieved either through its packing porosity or via the optimization of the vapor channel tortuosity. El Fil et al.^33^ have shown a 1.7× enhancement in sorption kinetics by creating vapor channels, thereby reducing tortuosity. In this work, we focus on coating thickness and overall water capacity to achieve higher amounts of daily water collection. Therefore, the key research point for lowering the diffusion resistance in coatings is to primarily optimize the diffusion thickness, which is directly affected by both the coating thickness and the tortuosity factor.

Figure 6C shows the instantaneous water uptake as a function of time. It highlights the effect of equilibrium uptake (*w*_eq_ = 0.25 and 1.00 g_water_ g_sorbent_^–1^), intracrystalline diffusivity (*D*_μ_ = 10^–17^and 10^–16^ m^2^ s^–1^), and coating thickness (δ_ads_ = 0.6 and 2 mm). At low uptakes (0.25 g_water_ g_sorbent_^–1^) and low kinetics (10^–17^ m^2^ s^–1^), the effect of the coating thickness is not significant. However, at higher uptake and kinetics (1.00 g_water_ g_sorbent_^–1^ and 10^–16^ m^2^ s^–1^), the coating thickness becomes the dominant factor. It is important to note that a random porous medium was assumed with a packing porosity of 0.5. With thicker coatings, less water can be adsorbed due to diffusion-limited vapor transfer through the coating thickness, as depicted by the regime map (Figure 6B). This favorable sorption performance reflects favorably on the total amount of daily water collected as shown in Figure 6D. For the same total amount of sorbent, the amount of water collected could increase by 1 order of magnitude due to the choice of sorbent and the sorbent bed geometry. For a sorbent with low equilibrium uptake of 0.25 g_water_ g_sorbent_^–1^ and intracrystalline diffusivity of 10^–17^ m^2^ s^–1^, the total amount of water collected for a sorbent layer of 0.6 and 2 mm is 133 and 71 mL, respectively. On the other hand, at higher uptakes and fast kinetics (1.00 g_water_ g_sorbent_^–1^ and 10^–16^ m^2^ s^–1^), the total amount of water collected for sorbent layers of 0.6 and 2 mm is 1340 and 396 mL, respectively.

In conclusion, a fin-array adsorbent bed design was proposed, offering rapid kinetics with high-density waste heat, enabled by a fin-array adsorbent bed design and millimeter thick adsorbent coatings for a compact form factor. The air gap between adsorbent fins was identified as a key design parameter to maximize water adsorption, optimized to ensure sufficient water vapor supply, and minimize mass transfer resistance. Overall, our strategy realizes scalable sorbent beds with ordered fin-array structures with synergistic enhancement of heat transfer and mass transport for enhancing the water sorption kinetics. We engineered and demonstrated a rapid-cycling SAWH prototype using a commercially available sorbent, i.e., AQSOA Z02. The prototype realizes fast water capture and desorption cycles with high yield water production of 5,826 mL_water_ kg_sorbent_^–1^ day^–1^.

## Experimental Procedures

### Numerical Simulation

A detailed numerical simulation is conducted using COMSOL Multiphysics version 6.0 for adsorption and desorption processes. For the adsorption process, a laminar flow is assigned to simulate the air flow within the millimeter air gaps. The adsorbent coating is modeled as a porous medium. The water vapor diffusion in air and the porous coatings is governed by the convection-diffusion equation, while adsorption of water vapor in the adsorbent crystals is modeled with linear driving force approximation. For the desorption process, the bottom surface is supplied with high-density waste heat. Slightly above the fin tip is a condenser connected with a heat sink. The vapor pressure at the condenser surface is fixed as the saturation pressure to simulate the condensation behavior. More detailed information is available in Note S1, Figures S1 to S4.

### Experimental Characterization

Adsorption and desorption characterization results are separately validated with the simulation data using 10 fins coated with zeolite Z02. A fan provides the humid air through the adsorbent bed, and the mass change is monitored during the adsorption process as the water uptake change. After the adsorption, the fins are placed in an enclosed system and heated by cartridge heaters imbedded in the base. The released water vapor is condensed and collected to measure the water desorption, and temperature profiles are recorded at different locations. More detailed information is available in Note S4, Figures S9 to S11.

## Data Availability

All data associated with the study are included in the article and the Supporting Information. Additional information is available from the Lead Contact upon reasonable request.

## Supporting Information Available

Additional information on adsorbent bed design, simulation, and experimental validation (Figures S1–S11 and Notes S1–S4) (PDF)
