# Supplementary material for: Design of a Compact Multicyclic High-Performance Atmospheric Water Harvester for Arid Environments
Source: ACS Energy Lett. 2024 Jun 26;9(7):3391–9. doi: 10.1021/acsenergylett.4c01061 (PMC11250079; doi:10.1021/acsenergylett.4c01061)
Supplement: Supplementary file 1 — nz4c01061_si_001.pdf [file nz4c01061_si_001.pdf]

Supporting Information

Design of a Compact Multicyclic High-performance Atmospheric Water Harvester for Arid Environments

Xiangyu Li[1,2†*], Bachir El Fil[1†*], Buxuan Li[1], Gustav Graeber[1,3], Adela C. Li[1], Yang Zhong[1], Mohammed Alshrah[1], Chad T. Wilson[1], Emily Lin[1]

[1] *Department of Mechanical Engineering, Massachusetts Institute of Technology, Cambridge, MA 02139, USA*

[2] *Department of Mechanical Aerospace and Biomedical Engineering, University of Tennessee Knoxville, TN 37996, USA*

[3] *Department of Chemistry, Humboldt-Universität zu Berlin, 12489 Berlin, Germany*

† Authors contributed equally to this work

* Correspondence, Email: xli148@utk.edu; belfil@mit.edu

Note S1: Numerical Simulation

A detailed numerical simulation of the adsorbent bed provides the toolset for device design and optimization. In this work, we used COMSOL Multiphysics v6.0 to develop thermal-fluidic models for different processes due to their distinctive physics: adsorption and desorption. To enable water adsorption at low humidities, we assumed a simplified Type-IV isotherm profile with a step function at 6% relative humidity (RH) with peak water uptake of 0.5 $g_{water}/g_{sorbent}$, as shown in Figure S1. The simplified isotherm also helps to illustrate the underlying physics of how the adsorbent bed design affects the adsorption kinetics for demanding arid regions. We assumed the desorption temperature of this material as 70 ˚C for desorption studies. The adsorbent materials

are then incorporated into a copper foam with a volume fraction of 50%, with an intercrystalline diffusivity of 6×10^{-6} m^2 s^{-1}, and intracrystalline diffusivity of 10^{-15} m^2 s^{-1} based on previous experimental work.[5,8,11] To ensure a wide range of deployment, we focus on an arid ambient condition of 10% RH at 27˚C, one of the most demanding conditions on earth for water harvesting.

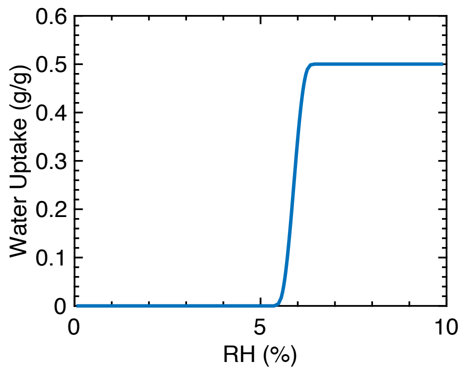

Figure S1 Simplified type-IV isotherm profile for adsorption numerical simulation model.

Figure S2 illustrates the adsorption bed design and adsorption process, where humid air flows through the gaps between adsorbent coatings. The adsorption process is modeled with modules of heat transfer, porous media, and transport of diluted species. Due to the periodic design, we can predict the overall adsorption performance with a 2D simulation within a single unit cell, as shown in Figure S2(a). A laminar flow profile is assigned to the air flow due to millimeter air gaps to minimize the computational cost (Figure S2(b)). As the humid air flows through the gaps between adsorbent coatings, the kinetics are dominated by the coating thickness, and total adsorbed water scales with the adsorbent fin height H. The adsorbent coating is modeled as a porous medium with 50% porosity and an effective thermal conductivity of 5 W/mK based on commercial copper foam properties. The water vapor diffusion in air and the porous coatings is governed by convection-diffusion equation, as shown in Eqn. E1,

$$\frac{\partial C}{\partial t} + u\nabla C = D_{\mathrm{v,air}}\nabla^2 C, \tag{E1}$$

where C is the water vapor concentration in air, u is air flow velocity, and $D_{\mathrm{v,air}}$ is the diffusion coefficient of water vapor in air, respectively. Inside the adsorbent coating, diffusion and adsorption of water vapor in the porous adsorbent coatings are modeled with a convection-diffusion equation and linear driving force approximation, listed as Eqn. E2 and E3,

$$\frac{\partial(\phi C)}{\partial t} = D_v \nabla^2(\phi C) - (1-\phi)\frac{\partial C_\mu}{\partial t}, \tag{E2}$$

$$\frac{\partial C_\mu}{\partial t} = \frac{15}{R^2} D_\mu (C_{eq} - C_\mu), \tag{E3}$$

where ϕ represents the porosity of the coating, D_v is the intercrystalline diffusivity in the coating determined by the coating porosity and tortuosity, D_μ is intracrystalline diffusivity governed by the adsorbent materials and the adsorbent crystal size R, C_μ is the water concentration in the adsorbent, and C_{eq} is the equilibrium water concentration in the adsorbent, as shown in Figure S2(c). Due to the adsorption enthalpy, mass transport is coupled with heat transfer as Eqn. E4,

$$\rho c_p \frac{\partial T}{\partial t} = k\nabla^2 T + h_{ad}(1-\phi)\frac{\partial C_\mu}{\partial t}, \tag{E4}$$

where ρ, c_p, k, T are the density, heat capacity, thermal conductivity and temperature of the adsorbent coating, h_{ad} is the adsorption enthalpy.

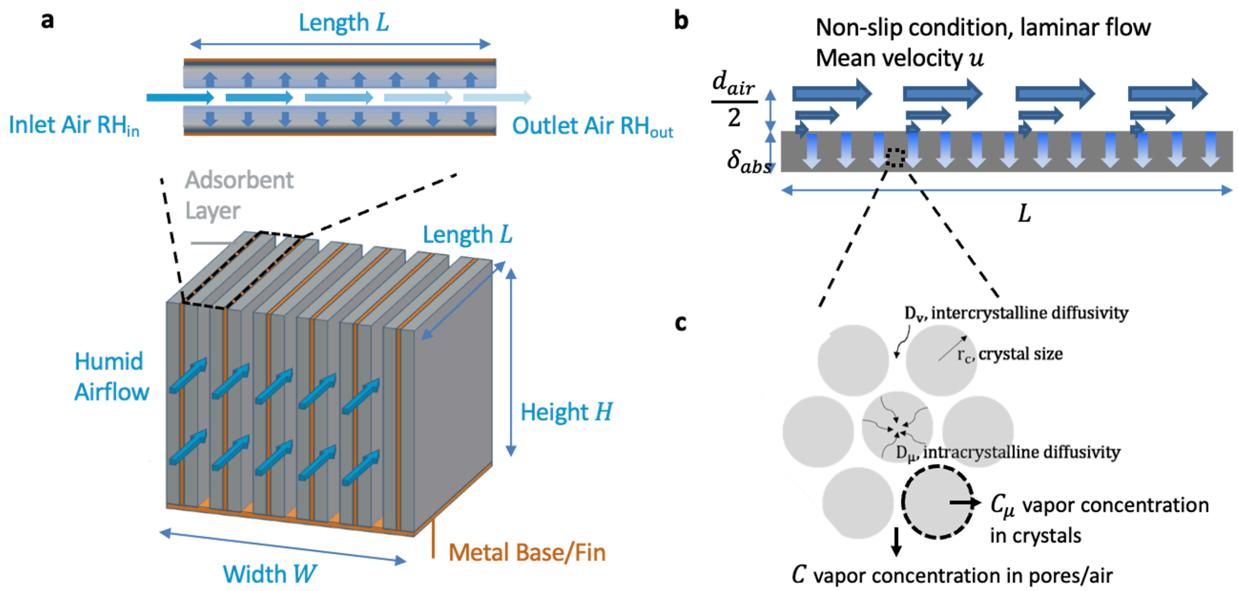

Figure S2 Adsorption numerical simulation model. (a) 2D representative unit for the adsorption process. As humid air flows through each air gap between coatings, the total adsorption scales with fin height H. (b) 2D simulation domain for the adsorption modeling. Half of the representative unit is constructed due to symmetry, with porous coating and linear driving model for adsorption. (c) Both intercrystalline and intracrystalline diffusivities are modeled.

Desorption occurs as waste thermal energy heats up the adsorption bed. The cross-section of repeated adsorbent fins is selected as the 2D simulation domain, as shown in Figure S3. A single adsorbent fin is enclosed within the air gaps. The bottom surface is supplied with either constant temperature or constant heat flux as the high-density waste heat. Slightly above the fin tip is a condenser connected with heat sink assuming an effective heat transfer coefficient of 800 W/m^{2}K. The vapor pressure at the condenser surface is fixed as the saturation pressure to simulate the condensation behavior.

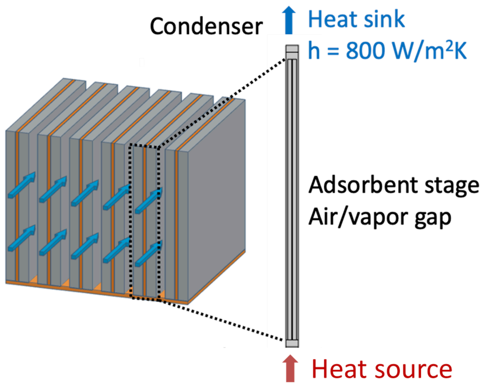

Figure S3 Desorption simulation model. Heat source is applied at the bottom surface as either constant temperature or constant heat flux, and total desorption scales with the channel length *L*. A condenser with heat sinks is included for vapor condensation.

To investigate the possible energy source for the proposed adsorbent bed, we investigated the effect of waste heat temperature on the desorption process, where 90˚C or higher heat sources show sufficient desorption kinetics. Therefore, we choose 90˚C as the constant temperature boundary condition. Besides constant base temperature as the boundary condition for the desorption process (Figure 4, Figure S4 (a)), other high density heat sources can be approximated as constant heat flux to drive the desorption process, as shown in Figure S4 (b). Choosing different boundary conditions can affect the desorption behaviors and dynamics. For constant temperature boundary condition, the desorption rate decreases with time, due to reduced temperature difference and heat flux between the heat source and adsorbent. As the desorption ends, the adsorbent reaches the base temperature. For constant heat flux boundary condition, the desorption rate remains constant after the initial diffusion process, due to the constant heat flux to overcome the adsorption enthalpy. Once most of the water is desorbed, the heat flux will continue to contribute to the sensible heating, overheating the adsorbent materials. In real-world applications, the boundary conditions can be more complicated than the two simplified scenarios, therefore it is critical to identify accurate boundary conditions to best leverage the high-density energy source for water desorption.

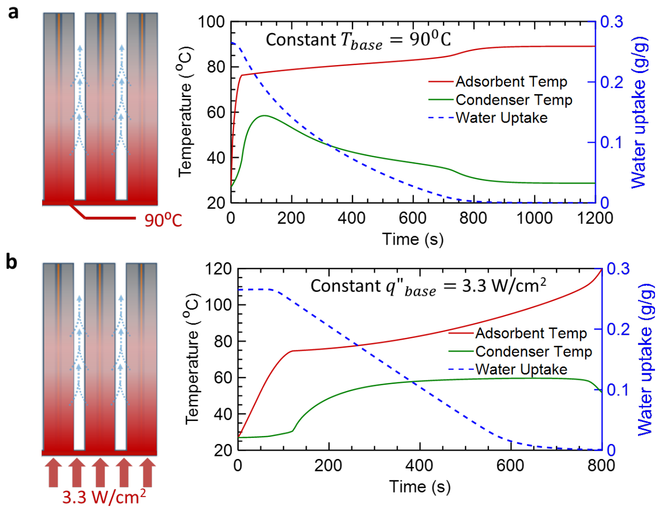

Figure S4 Desorption processes under (a) constant base temperature and (b) constant heat flux boundary conditions.

Note S2: Scaling analysis for outlet air flow relative humidity

Air flows through narrow gaps between adsorbent fins with a channel gap of d_{air}, therefore the mass transfer coefficient to the adsorbent coating scales with

$$h_m = D_{air} \cdot \frac{Sh}{d_{air}},$$

where d_{air} is the air gap thickness, Sh is a constant Sherwood number for internal laminar flow assuming fully developed flow, and D_{air} is the vapor diffusivity in air. The adsorbed water vapor flux can be scaled as $h_m\left(RH(x) - RH_{c,surf}\right)$, where $RH(x)$ represents the mean relative humidity along the channel length at position x, shown in Figure S5. Based on the mass conversation in the control volume,

$$RH(x + \Delta x)vd_{air} = RH(x)vd_{air} - 2h_m\,\Delta x\,(RH(x) - RH_{c,surf})$$

$$\frac{\Delta RH}{\Delta x} = -\frac{2h_m}{vd_{air}}\left(RH - RH_{c,surf}\right) = -\frac{2ShD_{air}}{d_{air}^2 v}\left(RH - RH_{c,surf}\right)$$

where RH is the relative humidity, v is the air flow velocity, $RH_{c,surf}$ is the relative humidity at the coating surface. Assuming constant $RH_{c,surf}$ during the early stage of the adsorption process,

$$RH(x) \sim \exp\left(-\frac{2ShD_{air}}{d_{air}^2 v}x\right) + RH_{c,surf}$$

Therefore, with constant D_{air}, L and $RH_{c,surf}$, the outlet RH scales with $d_{air}^2 v$.

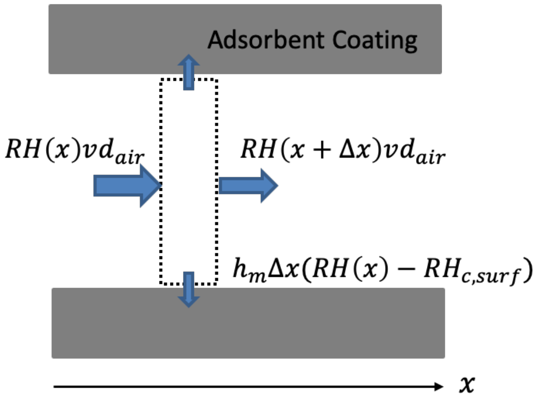

Figure S5 Control volume analysis of relative humidity in air channel flows

Note S3: Optimization of adsorbent bed design on adsorption and desorption processes

Table S1 Adsorption Bed Configuration

Adsorbent coating thickness δ_{abs}	0.6 mm
Metal fin thickness δ_{metal}	0.15 mm
Air gap thickness d_{air}	2 mm
Fin height H	4 cm
Air channel length W	3.5 cm
Air velocity	7 m/s
Peclet number	26.9

With 212 fins, the adsorbent bed expects to occupy a volume of

$$V = (N_{fin}(2\delta_{abs} + \delta_{metal} + d_{air}) + d_{air})HW = 997.1 \text{ mL}$$

Constant Base Temperature Boundary Condition

The adsorbent fin height is a design parameter in the adsorbent bed design. Since the adsorption process scales with the fin height, it only affects the desorption process, as illustrated in in Figure S6(a-c). Under constant temperature condition as shown in Figure S6(a), desorption thermal energy is supplied through thermal diffusion between base temperature and the desorption front. With higher fin height, the temperature gradient decreases, which reduces the heat flux and extends the desorption period, as indicated by in Figure S6(b, c). Here we assume that the desorption ends when water uptake reaches 0.01 g/g. Actual device operation may choose other threshold to end the desorption for higher desorption kinetics. On the other hand, the kinetics of adsorption and desorption processes are both affected with coating thickness, as shown in Figure S6 (d,e), assuming that a constant base temperature of 90ºC is applied as the waste heat. Within 1L volume adsorbent bed and 2 mm air gap between adjacent adsorbent fins (Table S1), thicker coatings have the benefits to pack more adsorbent materials, as shown in Figure S6 (f). However, thinner coatings with faster kinetics can overcome the smaller amount of adsorbent, to achieve higher amount of water adsorption in a sub-1hr adsorption cycle, resulting in higher daily water collection. We note that for practical applications, thinner coatings may also present larger fabrication challenges and overall cost.

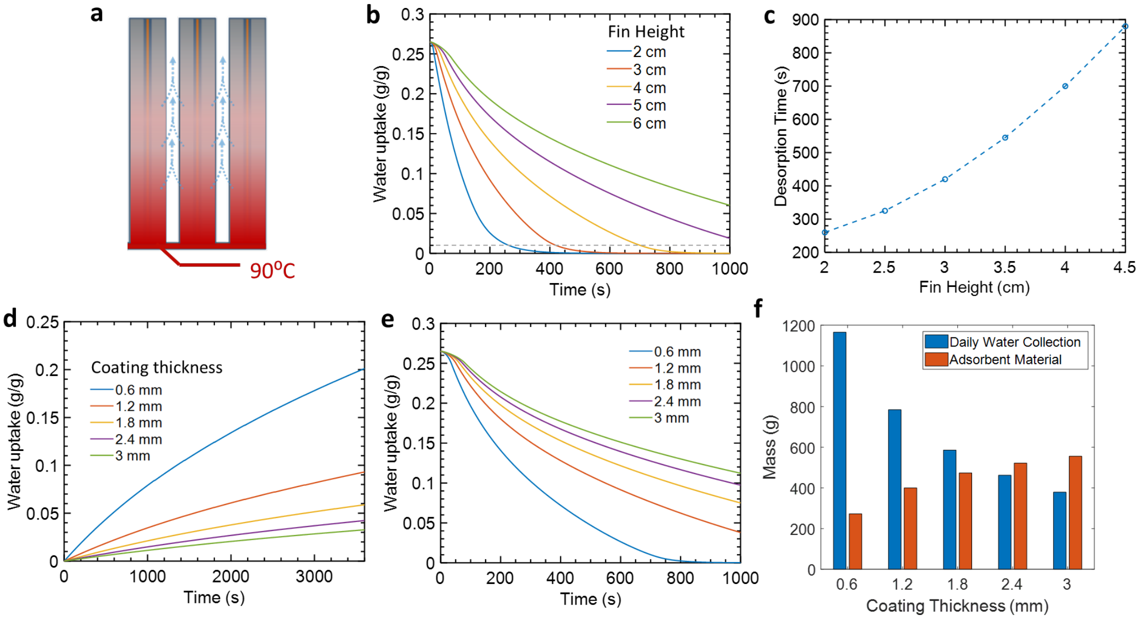

Figure S6 Effect of fin height and coating thickness. (a) Constant base temperature of 90ºC is applied for desorption process. Effect of fin height on the (b) water uptake during desorption process and (c) overall desorption period. Dashed gray line indicates the end of desorption with water uptake of 0.01 g/g. Effect of coating thickness on (d) adsorption, (e) desorption process, (f) total amount of adsorbent materials and daily water collection. We assume 1-hr adsorption-desorption cycle operation for all coatings.

Constant Heat Flux Boundary Condition

The effects of fin height and coating thickness on the desorption process are investigated with constant heat flux condition, as shown in Figure S7(a). As a constant thermal energy is supplied to the adsorbent materials to drive the desorption process, the desorption period increases linearly with the fin height, as shown in Figure S7 (b,c). Compared to Figure S6(e) where constant temperature condition is applied, the coating thickness has a much smaller impact on the desorption period, and the desorption time remains relative constant for thicker coatings, as shown in Figure S7(d). However, the dominant limiting factor of the total water production is still the adsorption kinetics, which occupies most of an adsorption-desorption cycle. Figure S7(e) illustrates the total adsorbent mass and daily water collection with 1L adsorbent bed, indicating a

similar trend, that thinner coatings are preferred due to much higher adsorption kinetics, contributed to the proposed fin-array adsorbent bed design.

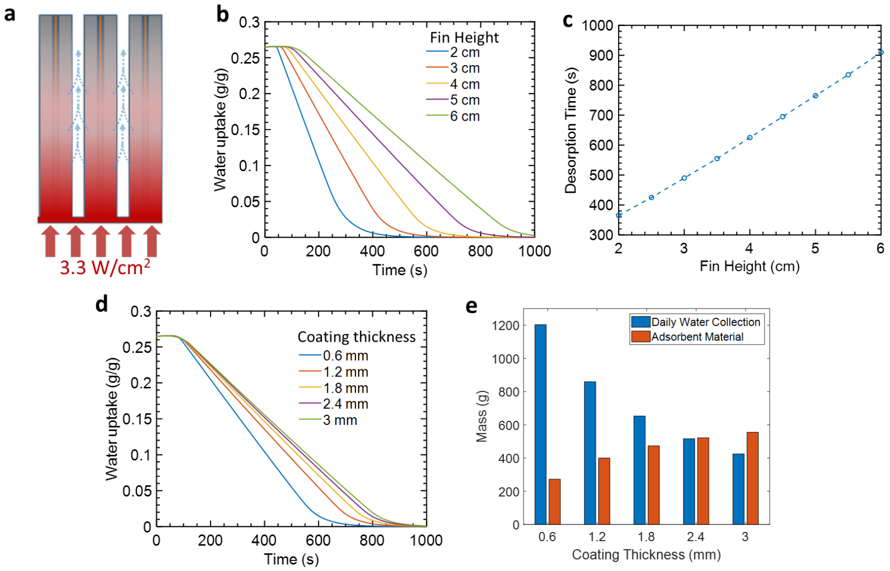

Figure S7 Effect of adsorbent fin height on the desorption process, under (a) constant heat flux boundary condition of 3.3 W/cm^2. (b,c) Under constant heat flux conditions, the desorption period increases linearly with the fin height, as the supplied thermal energy scales with higher amount of adsorbent materials with higher fins. (d) Desorption kinetics with different coating thicknesses. (e) The water collection and adsorbent materials are plotted based on various coating thickness, given 1L adsorbent bed volume.

Extending the fin height also increases the thermal resistance along the heat transfer direction, which leads to a higher base temperature especially with a high heat flux. With a constant heat flux boundary condition, the fin tip must reach desorption temperature for full desorption, when a significant temperature difference occurs with higher fins, as illustrated in Figure S8(a), governed by

$$\Delta T_{fin} = T_{base} - T_{tip} = q"H/k_{eff},$$

$$k_{eff} \approx (\delta_{metal}k_{metal} + 2\delta_{abs}k_{coating})/(2\delta_{abs} + \delta_{metal})$$

where $q"$ is the heat flux, H is the fin height, k_{eff} is the effective thermal conductivity of the fin along its height direction, k_{metal} and $k_{coating}$ are the thermal conductivity for metal fin and the

adsorbent coatings (porous metal foam and adsorbent powders). Figure S8(b) demonstrates the base temperature profiles (red lines) during the desorption for various fin heights, as well as the water uptake (blue lines).

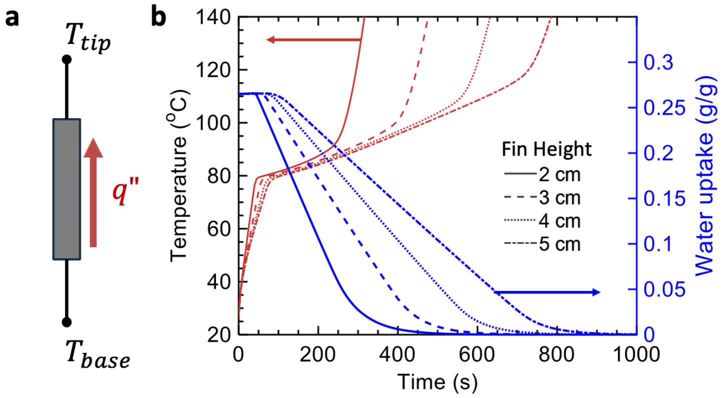

Figure S8 Effect of fin height on the fin base temperature during desorption process. (a) The heat flux boundary condition will introduce a temperature increase at the fin base for adsorbent to reach desorption temperature. (b) With higher fin height, the desorption demands higher base temperature to supply a constant heat flux at the base.

Note S4: Experimental Characterization

To validate the developed adsorption and desorption models, two setups were designed and built, for adsorption and desorption processes respectively, as shown in Figures S5, S6. The adsorption setup measures the water adsorption in the adsorbent bed. The desorption setup monitors temperature profiles and water collection. Additionally, the condensate liquid water is collected throughout the process. As a demonstration, each of the setups consists of 10 fins that are coated with zeolite Z02. The numerical models are updated with the corresponding uptake curves to take the type of adsorbent into consideration.

In the adsorption characterization setup, adsorbent fins are fixed on a copper plate and placed inside an acrylic square cross-section tube (50.8 mm × 50.8 mm). Using wire-electrical discharge machining (wire-EDM), 0.15 mm wide slits are machined into the copper base plate for the adsorbent fins to be inserted. The depth of these slits is 3 mm which is sufficient to hold the fins

securely onto the base plate. A fan is placed at the inlet of the tube to drive the humid air through the adsorbent bed, with controlled operating conditions (T, RH) at the inlet. The water uptake of the adsorbent is characterized by monitoring the mass change during the adsorption process. A top view of the adsorption testing setup is shown in Figure S9. Under ambient conditions in the lab, we observed fast saturation during a full cycle adsorption at about 2000 seconds. Each fin includes 1.2 g zeolite Z02.

Unlike the adsorption setup, the desorption/condensation setup is an enclosed system without active air flows. The copper plate is heated by cartridge heaters imbedded in the base. Initially, the adsorbent bed is left to adsorb water vapor in an environmental chamber at a known temperature and relative humidity. The base plate is heated to drive the desorption process. The released water vapor is then condensed on the surface of an anodized aluminum condenser. The measured temperature and mass of the condensate are compared with those of the numerical simulation. Heat sinks are connected to the condenser to help in rejecting the heat of condensation during the process, as shown in Figure S10(a). Figure S10(b) shows the setup insulated, instrumented, and mounted on top of the balance. Four thermocouples were connected to the setup measuring the base temperature, temperature of the center fin (mid-way and tip), and the condenser's surface temperature.

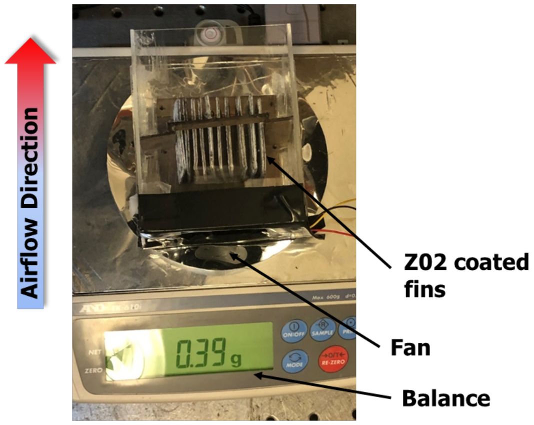

Figure S9 Actual image of the setup where the fan blows air over the Z02 coated fins. The adsorption setup is placed on a digital balance to measure the real-time change of mass during the adsorption process.

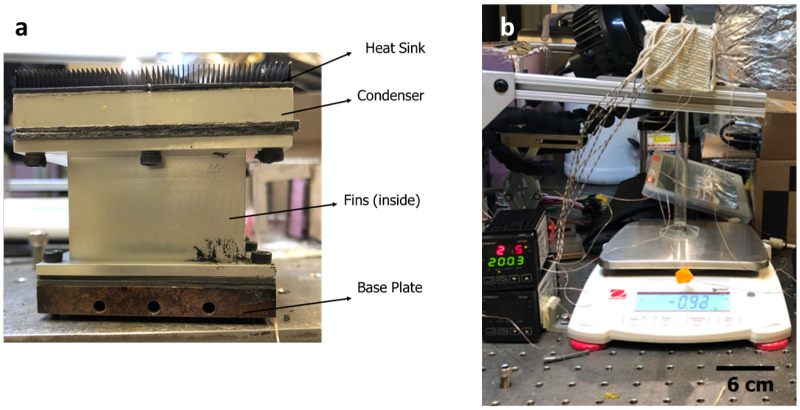

Figure S10 The desorption-condensation characterization setup. (a) A zoomed-in image of the setup showing the heat sink, condenser, aluminum walls holding the Z02 coated fins within, and the base plate. (b) Image showing the mounting of the desorption-condensation setup connected to the heater and digital balance to measure the amount of water condensed.

The temperature of the base was controlled and set as 200 °C. Water collection started when the temperatures of the fin and base were ~100 °C and 145 °C, respectively. However, we noticed as steady state was achieved, the measured temperature of the fins was slightly lower than the modeling due to extra heat loss during the experiments. The modeling domain assumes that the

fins experience adiabatic boundary conditions on the sides. We quantified the heat loss during our experiments to be about 8.8 W, which corrected for the mismatch of the fin temperature profiles.

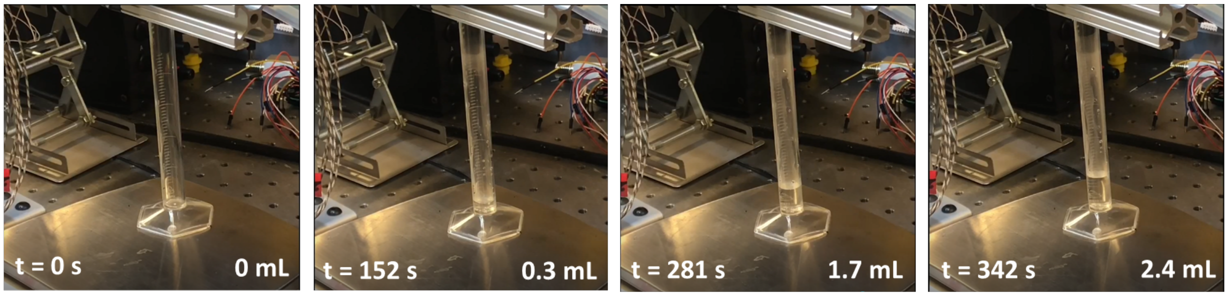

Figure S11 A timelapse showing the amount of water collected during the desorption process.
